# Age at first exposure to antibiotics and neurodevelopmental outcomes in childhood

**DOI:** 10.1007/s00213-023-06351-5

**Published:** 2023-03-17

**Authors:** Rebecca F. Slykerman, Denise Neumann, Lisa Underwood, Mark Hobbs, Karen E. Waldie

**Affiliations:** 1 Department of Psychological Medicine, Faculty of Medical and Health Sciences, University of Auckland, Building 507, 22-30 Park Avenue, Grafton, Auckland, 1023 New Zealand; 2 School of Psychology, Faculty of Science, University of Auckland, Auckland, New Zealand; 3 Growing UP in New Zealand, Centre for Longitudinal Research, University of Auckland, Auckland, New Zealand

**Keywords:** Antibiotics, Cognitive development, Behaviour

## Abstract

**Rationale:**

Viral illnesses in children are common and are frequently treated with antibiotic medication. Antibiotics reduce the diversity and composition of the gut microbiota, leading to poor developmental outcomes.

**Objectives:**

To investigate the relationship between age at first exposure to antibiotics and cognitive and behavioural development at 4.5 years while controlling for multiple confounders, including otitis media.

**Methods:**

Study participants were 5589 children enrolled in the broadly generalisable Growing Up in New Zealand cohort study, with antibiotic exposure data, maternal antenatal information, and age 4.5-year behaviour and cognitive outcome data. Children were categorised as first exposed to antibiotics according to the following mutually exclusive ages: 0–2 months; 3–5 months; 6–8 months; 9–11 months; 12–54 months or not exposed by 54 months. Developmental outcome measures included the Strengths and Difficulties Questionnaire, Luria hand clap task, and the Peabody Picture Vocabulary Test-III.

**Results:**

In univariate analysis, there was an evident dose–response relationship where earlier exposure to antibiotics in the first year of life was associated with behavioural difficulties, lower executive function scores, and lower receptive language ability. After adjusting for confounders, pairwise comparisons showed that first antibiotic exposure between birth and 3 months or between 6 and 9 months was associated with lower receptive vocabulary. Antibiotic exposure at any age prior to 12 months was associated with increases in behavioural difficulties scores at 4.5 years.

**Conclusions:**

Following adjustment for socioeconomic factors and otitis media, there is evidence that antibiotic exposure during potentially sensitive windows of development is associated with receptive language and behaviour later in childhood.

## Introduction

The remarkable development of the central nervous system that begins in the prenatal period and extends throughout childhood underpins cognitive development and establishes a trajectory of continued typical cognitive skill acquisition. Alongside this growth and specialisation of the brain, establishing a stable gut microbial colony in the gastrointestinal tract occurs. A series of complex systems that regulate physiological function and connect the gut and the brain, known as the gut-brain-axis, develop to set the physiological scene for future health. During the development of these organ and regulatory systems, during sensitive periods, development is heavily influenced by external factors that alter the composition of the gut microbiota (Cowan et al. 2020). The development and stabilisation of the infant gut microbiota appear to follow a biological plan. However, early life factors, including mode of delivery, feeding practices, and antibiotic exposure, influence early microbiota development (Korpela and de Vos 2018).

Viral illnesses in children are common, and many of these occur in preschool children and result in high prescription antibiotic medication use (Rogawski et al. 2017). In a New Zealand cohort of children born in the mid-1990s, approximately 70% of children had received antibiotics in their first year of life (Slykerman et al. 2017), and by the age of five years, nearly all children have received antibiotics as a treatment for these common illnesses, with amoxicillin the most frequently prescribed antibiotic (Hobbs et al. 2017). Antibiotics reduce the diversity in the gut microbiota and alter the taxonomic composition of the microbiome, and this reduction in the richness of the microbes is considered detrimental to health (McDonnell et al. 2021; Ramirez et al. 2020). A single course of antibiotics can alter the infant gut microbiota permanently. In a study of infants prescribed amoxicillin, the gut did not return to pre-antibiotic composition but began an accelerated course towards a more mature gut composition with low *Bifidobacterium* abundance. The average age of infants in this study was 4 months, an age when *Bifidobacteria* would be the dominant strain in healthy breastfed infants before weaning (Korpela et al. 2020).

The development of multidirectional connections linking the gut microbiota and the central nervous system during infancy and childhood provides a mechanism by which early-life exposure to antibiotics might influence developmental outcomes. Investigation of this association in human populations relies on observational studies, given that it would not be ethical to conduct a randomised controlled trial of antibiotic prescription for illness in children. Longitudinal cohort studies from birth throughout childhood, with both antibiotic and developmental information, provide an opportunity to examine associations between antibiotic exposure and neurodevelopment. Our first report on this topic showed an association between antibiotic exposure in the first year of life and detrimental behavioural, emotional, and cognitive outcomes compared with children who received their first antibiotic exposure after 12 months of age (Slykerman et al. 2017). This finding was confirmed in a subsequent study using a different cohort whereby children who received antibiotics in the first 6 months of life had significantly lower verbal and overall cognitive ability scores at age 11 on psychologist-administered intelligence tests and increased problems with executive function, including metacognitive abilities, impulsivity, attention, and anxiety (Slykerman et al. 2019). Long-term antibiotic use in childhood is associated with an increased risk of anxiety and depression, with findings suggesting that gene interactions with chronic antibiotic exposure in childhood increase the risk of mood disorders later in life (Liang et al. 2021).

Small sample sizes and retrospective parent reports of antibiotic use are limitations of previous studies. A consistent criticism of investigations of the association between antibiotic exposure and neurodevelopmental outcomes has been the inability to control for the confounding effects of otitis media. Chronic ear infections are associated with antibiotic treatment and delayed language development (Schilder et al. 2016), making them a primary potential confounder in observational studies of the relationship between antibiotics and neurodevelopment. Furthermore, early childhood development is heavily influenced by the quality of the environment in which the child develops. Adequate adjustment for the effects of early environment on outcomes is difficult to achieve, and it is possible that residual confounding could explain the previously reported associations between antibiotic exposure and poorer cognitive and behavioural outcomes later in childhood.

We embarked on the current study to address some of the limitations of previous investigations. We aimed to advance understanding of the role of early antibiotic use in development by utilising a large cohort of preschool children, antibiotic dispensing data, a comprehensive measure of socioeconomic environment, and adjustment for otitis media. Specifically, this study aimed to investigate the relationship between age at first exposure to antibiotics and later cognitive and behavioural development at 4.5 years. We hypothesized that antibiotic exposure in infancy would be associated with an increased risk of atypical scores on neurodevelopmental outcome measures. Further we hypothesized that younger age at first exposure to antibiotics would be associated with the greatest risk of abnormal cognitive and behavioural scores at 4.5 with this risk decreasing as age at first exposure increased.

## Methods

### Participants

Study participants were enrolled in the longitudinal, *Growing Up in New Zealand* cohort study. The study’s design and recruitment procedure have been previously described (Morton et al. 2012). Briefly summarised, the study’s cohort comprises a socioeconomically and ethnically diverse sample of children born to 6822 pregnant women from three contiguous District Health Board regions in New Zealand who had expected delivery dates between 25 April 2009 and 25 March 2010. The cohort is broadly generalisable to current New Zealand births (Morton et al. 2015). Significant data collection waves when the children were 9 months, 2 years, and 4.5 years of age have included conducting computer-assisted telephone and personal interviews to gather information relating to six inter-connected domains of child development: health and wellbeing; cognitive and psychosocial; education; family and whānau (extended family); culture and identity; and neighbourhoods and societal context.

The Ministry of Health Northern Y Regional Ethics Committee (NTY/08/06/055) approved the study, and mothers provided written informed consent for their own and their children’s participation. Primary caregivers provided informed consent for linkage to routinely collected administrative health data using each child’s National Health Index (NHI) number, a unique alphanumeric identifier used within the New Zealand health system. Data sources used included perinatal health records held by maternity carers and birth hospitals and community antibiotic dispensing data held in the Pharmaceutical Collection.

A total of 5665 children had antibiotic exposure data up to 4.5 years of age. Up to 5589 children (98.7% of the sample) were included in the analyses for the current study, if their mothers provided antenatal information and if they had behaviour and cognitive outcome data at the 4.5-year follow-up. The number of children included in the univariate and multivariable analyses for the study ranged from 5098 (Luria multivariable analysis) to 5586 (univariate behaviour outcomes analysis). The smallest group for analysis was 5098. Children included in this analysis had a higher gestational age (*p* = 0.037), were less likely to have a low birthweight and more likely to have a high birthweight (*p* < 0.001), less likely to have had an assisted birth (*p* < 0.001), and more likely to be female (*p* = 0.01). Additionally, their mothers were more likely to be ≥ 30 when they were born (*p* < 0.001), more likely to be European and less likely to be a Māori or Asian mother (*p* < 0.001), and more likely to live in a low or medium deprivation household (*p* < 0.001) than those with missing data (*n* = 567; 10% of the sample).

### Antibiotic exposure

The Pharmaceutical Collection includes a record of all prescription medicines dispensed where a community pharmacist has sought government subsidisation (Ministry of Health 2015). It does not record hospital inpatient dispensing or medications dispensed directly by a medical practitioner in an emergency. Data were analysed only for antibiotics for systemic use. We excluded topical preparations and antifungal, antiviral, or antiparasitic medications. In New Zealand, antibiotics for systemic use are only available with a prescription, and antibiotics are dispensed free of charge for children under the age of 13 years (Ministry of Health 2018). The primary exposure variable was the age children were first exposed to antibiotics. Proposed sensitive windows exist for the parallel development of the gut microbiota and the brain and for the gut brain axis that connects these systems (see Cowan et al. 2020 for a review). Based on the premise that environmental influences such as antibiotic exposure are likely to differentially influence development and the high degree of granularity in age at first antibiotic exposure in our sample, children were categorised as first exposed to antibiotics according to the following mutually exclusive age ranges: 0–2 months, 3–5 months, 6–8 months, or 9–11 months. The reference group was those children who had their first exposure at 12–54 months or who had not been exposed to antibiotics by the age of 54 months.

### Outcome measures

#### Behaviour problems

We measured behaviour problems using the parent-report Strengths and Difficulties Questionnaire (SDQ) for ages 4–16 at 4.5 years (Goodman 1997). This 25-item questionnaire is widely used to screen for behavioural difficulties in childhood and is a valid measure of behaviour problems in the preschool population (Croft et al. 2015; D'Souza et al. 2017; Warnick et al. 2008). There were 5586 children (98.6% of the sample) with SDQ data.

#### Cognitive outcomes

A modified version of Luria’s pencil tapping task from the Luria-Nebraska neuropsychological battery (Golden et al. 1980) measured control. The task involves reverse imitation, requiring the child to hold arbitrary rules in working memory and inhibit a natural response to execute a pre-determined motor act (Putko and Złotogórska 2014). The child must provide the opposite response to interviewers’ modelled clapping (i.e., when the interviewer clapped once, the child must clap twice and vice versa). The task is a sensitive measure of executive control, especially among younger children (Putko and Złotogórska 2014). There were 16 hand clap test trials, with children receiving 1 point per trial for correct execution of the required action, adding up to a maximum score of 16. In the current study, the total Luria performance score was converted into a *z* score and then dichotomised into one standard deviation or more below the mean as below average executive control and all others as typical executive control performance (Buckley et al. 2020). There were 5201 children (91.8% of the sample) with Luria data.

Children’s receptive language ability was assessed using the Peabody Picture Vocabulary Test (PPVT-III) (Dunn & Dunn 1997). This short adapted version is based on a measure developed by the *Longitudinal Study of Australian Children* (LSAC) (Taylor et al. 2013). The PPVT is an individually administered norm-referenced test of single-word receptive vocabulary. It includes three sets of items—the core, basal, and ceiling set (20 items each). The core set was administered to all children, and if children made fewer than seven errors, they were given the ceiling set. The test ended if the children made between 7 and 14 errors. If the children made more than 14 errors, children were administered the basal set. Children received a raw score by adding correctly identified pictures between basal and ceiling items. We calculated PPVT-III scores by submitting the total number of correct responses to an item-response theory factor analysis. The final factor analysis adjusted PPVT-III score ranged from 0 to 40, consistent with the original scale. Those who scored one standard deviation or more below the mean were categorised as having receptive language performance below average and all others as showing typical receptive language performance. There were 5351 children (94.5% of the sample) with PPVT-III data.

### Covariates

Birthweight was categorised into three groups: low birthweight (< 2500 g), average birthweight (2500–4000 g), and high birthweight (> 4000 g). Gestational age was a continuous measure in analyses. Mode of delivery was dichotomised into spontaneous vaginal birth or assisted birth (comprised of planned caesarean, emergency or unplanned caesarean, or other assisted birth). At the 2-year data collection wave, mothers reported how many ear infections their child had suffered since the age of 9 months (never, 1–3, 4, or more).

Demographic factors included in the adjusted analysis were the child's sex, maternal age at birth of the child, and mothers’ externally prioritised ethnicity according to Statistics New Zealand prioritisation guidelines: Māori, Pacific Peoples, Asian, Middle Eastern/Latin American/African (MELAA), and Other (MELAA and Other combined for analysis due to small sample size).

The New Zealand Deprivation Index (NZ Dep) measures socioeconomic status. Information from the 2006 census identifies areas known as mesh blocks that are given a decile value ranging from 1 (least deprived) to 10 (most deprived) (Salmond et al. 2007). In the current study, deprivation was categorised into high (deciles 8–10), medium (deciles 4–7), and low (deciles 1–3).

### Statistical analysis

Statistical analysis was conducted in 25.0 IBM SPSS Statistics. Logistic regression analyses examined the relationship between antibiotic exposure and cognitive outcomes at age 4.5. Linear regression examined the association between age at antibiotic exposure and behavioural difficulties. Multivariable regression analyses examined the relationship between antibiotic exposure and the outcome variables adjusted for potential confounders.

## Results

Table 1 shows the number and percentage of children in this sample who had their first exposure to antibiotics in each age group. Table 2 describes the characteristics of our sample.

**Table 1:** Age at first exposure to antibiotics

Antibiotic exposure	*n* (%)
0 < 3 months	485 (8.6%)
3 < 6 months	886 (15.6%)
6 < 9 months	1180 (20.8%)
9 < 12 months	954 (16.8%)
12 < 54 months or not exposed by 54 months	2160 (38.1)
Total	5665

**Table 2:** Characteristics of the study sample

Covariates		*n* (%)
Birthweight (*n* = 5553)	Low birthweight (< 2500 g) Normal birthweight (2500–4000 g) High birthweight (> 4000 g)	234 (4.2%) 4382 (78.9%) 937 (16.9%)
Gestational age (*n* = 5512)	Mean (standard deviation)	39.18 (1.71)
Mode of delivery (*n* = 5535)	Spontaneous vaginal birth Assisted birth	3658 (66.1%) 1877 (33.9%)
Child sex (*n* = 5591)	Female Male	2702 (48.3%) 2889 (51.7%)
Maternal age at child’s birth (*n* = 5519)	< 20 years 20–29 years ≥ 30 years	225 (4.1%) 2060 (37.3%) 3234 (58.6%)
Maternal ethnicity (*n* = 5990)	European Māori Pacific Peoples Asian Other	3054(51.0%) 1066 (17.8%) 791 (13.2%) 869 (14.5%) 210 (3.5%)
NZ socioeconomic deprivation (*n* = 5590)	High (deciles 8–10) Medium (deciles 4–7) Low (deciles 1–3)	2016 (36.1%) 2091 (37.4%) 1483 (26.5%)
Number of ear infections between 9 months and 2 years of age (*n* = 5960)	None 0–3 4 or more	3114 (50.7) 2116 (34.4) 730 (11.9)

Of the children included in our analyses, 1055 children (20.3%) showed a performance below average in the executive control task, and 1160 children (21.7%) performed below average in their receptive language (Table 3).

**Table 3:** Bivariate and multivariate regression analyses results for the association between timing of first exposure to antibiotics and behavioural and cognitive outcomes at 54 months

	Behavioural difficulties (SDQ) *N* = 5586		Executive control (Luria task) *N* = 5201		Receptive vocabulary (PPVT) *N* = 5351	
*n* (%)			Typical: 4146 (79.7%)		Typical: 4191 (78.3%)	
			Low: 1055 (20.3%)		Low: 1160 (21.7%)	
Antibiotic exposure	Unadjusted estimate (95%CI)	Adjusted* estimate (95%CI)	Unadjusted OR (95%CI)	Adjusted* OR (95%CI)	Unadjusted OR (95%CI)	Adjusted* OR (95%CI)
0 to < 3 months	2.31 (1.80–2.82)	0.93 (0.43–1.43)	1.53 (1.21–1.94)	1.24 (0.96–1.60)	2.24 (1.78–2.81)	1.33 (1.02–1.74)
3 to < 6 months	1.68 (1.27–2.08)	0.45 (0.05–0.86)	1.16 (0.95–1.42)	0.92 (0.74–1.15)	1.62 (1.34–1.97)	1.09 (0.87–1.36)
6 to < 9 months	1.13 (0.77–1.50)	0.39 (0.03–0.75)	0.96 (0.80–1.16)	0.89 (0.73–1.09)	1.54 (1.28–1.84)	1.29 (1.05–1.58)
9 to < 12 months	0.80 (0.41–1.19)	0.55 (0.17–0.93)	0.95 (0.78–1.16)	0.90 (0.73–1.12)	1.24 (1.02–1.51)	1.20 (0.96–1.50)
12 to < 54 months or not exposed by 54 months	REF	REF	REF	REF	REF	REF

*Adjusted for maternal age, maternal ethnicity, socioeconomic status, mode of delivery, child sex, birthweight, gestational age, and otitis media

Table 3 shows the univariate and multivariable regression results for age at first antibiotic exposure and the three developmental outcomes of interest. In univariate analysis, antibiotic exposure was not significantly associated with atypical executive function scores at age 4.5 years. A dose–response relationship was evident in the bivariate analysis of the association between age at first exposure to antibiotics and behavioural difficulties and receptive language ability at 4.5 years. Earlier exposure to antibiotics within the first year of life was associated with an increased risk of atypical language development. For example, children who received antibiotics before 3 months of age were 2.24 times more likely to have below-average receptive language scores than children who first received antibiotics at 12 months or older. After adjustment for confounders, the overall association between antibiotic exposure and receptive language scores (*p* = 0.07) was not statistically significant. However, pairwise comparisons indicated that first exposure to antibiotics before the age of 3 months was associated with an increased risk of receptive vocabulary delay (OR = 1.34. 95%CI: 1.02, 1.74). First exposure to antibiotics between 6 and 9 months was also associated with an increased risk of receptive vocabulary delay (OR = 1.29. 95%CI: 1.05, 1.58). For behavioural difficulties, exposure to antibiotics at any of the age groups prior to 12 months of age was associated with small but statistically significant increases in behavioural difficulties score at 4.5 years. Children with first exposure to antibiotics between birth and 3 months had total difficulties scores an average of 0.93 points higher (95%CI: 0.43, 1.43) than those first exposed to antibiotics after the first year of life (Table 3).

In adjusted analysis, otitis media was significantly associated with inhibitory control (*p* = 0.03), but not with behavioural outcomes (*p* = 0.99) or receptive language (*p* = 0.16) (data not shown).

## Discussion

In this large population-based cohort study, bivariate analysis showed that earlier first exposure to antibiotics in infancy was associated with poorer cognitive and behavioural outcomes, with an evident dose–response relationship. However, after adjustment for a range of potential confounders, including otitis media, this dose–response relationship was less clear. Pairwise comparisons showed an increased risk of receptive language problems in children first exposed to antibiotics under the age of three months. Children who first received antibiotics in any of the age groups before 12 months of age had elevated behavioural difficulties scores. This is consistent with previous studies that have reported small but statistically significant associations between antibiotic exposure and lower cognitive ability scores in adjusted analyses (Slykerman et al. 2019, 2017).

Previous studies have reported significant associations between the microbial composition of the gut, which can be influenced by antibiotic exposure and emotional development. A prospective study of over 300 infants found that gut microbiota composition was associated with infant temperament. Reduced alpha diversity was correlated with increased fear reactivity and negative reactivity, while a microbial composition rich in *Bifidobacterium* was associated with positive emotionality (Aatsinki et al. 2019). The commonly prescribed antibiotic amoxicillin reduces *Bifidobacterium* abundance in infants, leading to an accelerated maturation of the gut composition (Korpela et al. 2020). In an Australian cohort of 201 children, decreased *Prevotella* abundance at 12 months was associated with greater internalising problems at two years of age, and antibiotic exposure was the best predictor of *Prevotella* abundance (Loughman et al. 2020).

Prior observational studies have not accounted for the potentially confounding effect of otitis media on early behaviour and language development in childhood. Chronic otitis media has been associated with antibiotic use to treat the infection and with lower language scores and increased behavioural difficulties (Da Costa et al. 2018; Niclasen et al. 2016; Schilder et al. 2016). It is possible that by adjusting for otitis media in analysis, the current study has reduced residual confounding present in earlier reports, yet there remains evidence in the current study that early exposure to antibiotics during potentially sensitive windows is associated with receptive language and behavioural difficulties. Interestingly, otitis media was not significantly associated with either behaviour or receptive language in our cohort indicating it is not a strong predictor of these developmental outcomes.

Given the ethical constraints on randomising children to receive antibiotic treatment, it is difficult to conduct the theoretically ideal study to elucidate the contribution of infection itself in the gut brain axis and to distinguish whether infection or antibiotic use may result in poorer neurodevelopmental outcome. We cannot rule out the possibility that antibiotic-treated infections were more severe and there is a possibility that infection itself contributed to neurodevelopmental outcome. Otitis media has been associated with lower language scores with the proposed mechanism that recurrent or chronic ear infections affect hearing during critical periods of language development. However, common childhood infections that occur as part of typical development and are important for development of acquired immunity are not known to cause long term cognitive deficits. Furthermore, if those children who received antibiotics did in fact have more severe infections, and more severe infections were in turn associated with developmental outcome, our results would have been more likely to show a significant overall correlation between antibiotic use and developmental outcome.

Early language, inhibitory control, and behavioural development are complex cognitive skills that are influenced by multiple genetic and environmental factors, presenting a challenge when attempting to control for these factors in observational studies. In particular, much has been written about the observed association between the socioeconomic status of the family and cognitive outcomes in children. Socioeconomic status is generally considered to capture a variety of factors including, but not limited to, parental education, income, and relationship status (Letourneau et al. 2013). The pathways through which the economic status of the family may influence developmental outcomes include the quality of parent–child interaction, access to enriching resources, and individual child and parent factors (Pace et al. 2017). Previous studies examining the relationship between antibiotics and development have used parental occupation, income, maternal age, and maternal education to adjust for socioeconomic status (Slykerman et al. 2019, 2017). In the current study, we used a comprehensive marker of economic deprivation that also takes into account resources at a neighbourhood and community level. By using this multidimensional economic deprivation variable in analysis, the current study is likely to have further reduced residual confounding that may have been present in earlier reports. Alternatively, it is also possible that using a comprehensive measure of socioeconomic deprivation has resulted in over adjustment of a factor operating as part of the causal pathway between antibiotic use and development. Children from low socioeconomic status backgrounds may also be more susceptible to infections resulting in antibiotic prescription. By using a multidimensional socioeconomic variable, it is possible that the reduction in magnitude of risk associated with earlier antibiotic exposure may have obscured the dose response relationship between age at first exposure and the outcomes in adjusted analysis. Controlling for socioeconomic status while not over-adjusting for a factor that may be on the causal pathway is a challenge in observational studies such as this one (Schisterman et al. 2009).

Our study has some limitations. We did not measure the microbial composition in infants in the cohort. Therefore, we cannot describe the effect of antibiotics on infant gut composition or the relationship between gut composition and developmental outcome. The majority of children receiving antibiotics were prescribed amoxicillin or amoxicillin clavulanate (> 70%) (Hobbs et al. 2017). Therefore, it was not possible to examine the associations between specific antibiotics and developmental outcomes, and future studies using large cohorts or registry data may be able to investigate the role of different antibiotic strains in the gut brain axis. The measurement of otitis media was reliant on accurate retrospective recall. Parents were asked when their child was 2 years old, how many ear infections the child had experienced since the age of 9 months. Some parents may have inaccurately recalled the exact number of infections, leading to an increase in measurement error and making it less likely we would detect a relationship between otitis media and language development. Data were collected in New Zealand, which has a specific mixture of ethnic and migration patterns; therefore, generalising to other populations should be done cautiously.

The study has several methodological strengths. The Growing Up in New Zealand cohort provides a large, socioeconomically, and ethnically diverse sample, which is a strong representation of the general population of contemporary New Zealand. The prospective longitudinal study design is another key strength, as it allowed us to gather data on antenatal factors without being subject to significant recall bias. Antibiotic data from dispensing records allowed us to determine how old each child was when they were first exposed to antibiotics with a high degree of granularity. Lastly, children’s cognitive ability was assessed using an objective, individually-administered test, rather than relying solely on parent or teacher reports which can be subject to bias because they are also influenced by the rater’s own cognitive and emotional process (De Los Reyes and Kazdin 2005).

In conclusion, we found evidence of a significant association between exposure to antibiotics and increases in behavioural difficulties scores. Further, antibiotic exposure in the first 3 months of life or between 6 and 9 months was associated with lower receptive language skills at 4.5 years. Our results suggest that future studies using registry data or existing longitudinal birth cohort studies will contribute to understanding the nature of the relationship between antibiotics and early development.

## Data Availability

Data for this manuscript is subject to ethical and confidentiality restrictions.
